# *Aralia taibaiensis* Protects against I/R-Induced Brain Cell Injury through the Akt/SIRT1/FOXO3a Pathway

**DOI:** 10.1155/2019/7609765

**Published:** 2019-05-12

**Authors:** Jialin Duan, Jia Cui, Hongnan Zheng, Miaomiao Xi, Chao Guo, Yan Weng, Ying Yin, Guo Wei, Jinyi Cao, Yanhua Wang, Aidong Wen, Boling Qiao

**Affiliations:** ^1^Biomedicine Key Laboratory of Shaanxi Province, College of Life Science, Northwest University, Xi'an 710069, China; ^2^Department of Pharmacy, Xijing Hospital, Fourth Military Medical University, Xi'an 710032, China; ^3^Department of Natural Medicine, School of Pharmacy, Fourth Military Medical University, Xi'an 710032, China; ^4^School of Basic Medicine and Clinical Pharmacy, China Pharmaceutical University, Nanjing 211198, China

## Abstract

**Background:**

Saponin from Aralia taibaiensis (sAT) showed excellent antioxidative effects in several models; however, its effects on brain cells were unknown to us. The present study was designed to evaluate the protective effects of sAT on ischemia/reperfusion- (I/R-) induced injury and clarify its mechanisms.

**Methods:**

*In vitro*, HT22 cells were pretreated with sAT and then subjected to I/R. Apoptosis rate, mitochondrial function, and antioxidant proteins were measured. To clarify the mechanisms, siRNA were used. *In vivo*, sAT was pretreated through intragastric administration for 7 days and the I/R model was induced. The neurobehavioral scores, infarction volumes, and some cytokines in the brain were measured. Protein levels were investigated by Western blotting.

**Results:**

The results showed that sAT treatment significantly protected cells from I/R-induced cell apoptosis and mitochondrial dysfunction. The antioxidant protein levels were increased in a dose-dependent manner. Further study revealed that sAT induced the deacetylation and phosphorylation of PGC-1*α* and FOXO3a. sAT treatment also induced the phosphorylation levels of Akt and the expression levels of SIRT1. Using the specific targeted siRNA transfection, the interplay relationship between Akt, SIRT1, PGC-1*α*, and FOXO3a was verified. Furthermore, the same protective effects were also observed in rats subjected to I/R.

**Conclusion:**

sAT protected brain cells from I/R-induced mitochondrial oxidative stress and dysfunction through regulating the Akt/SIRT1/FOXO3a/PGC-1*α* pathway.

## 1. Introduction

Ischemic stroke which always leads to disability and high mortality is one of the leading causes of death [1]. The occlusion of vessel induces ischemic injuries, and the reperfusion aggravates pathological changes such as oxidative stress, inflammation, calcium overload, glutamate excitotoxicity, mitochondrial dysfunction, and apoptosis; this process is also known as ischemia/reperfusion (I/R) [2–5]. Clinically, thrombolytic agents are the commonly used drugs in treating stroke, but the arrow time window limits its application [6]. Thus, searching novel therapeutic agents for ischemic stroke treatment is very necessary and urgent.

The brain is particularly vulnerable to oxidative stress. In normal cells, reactive oxygen species (ROS) are produced in mitochondria and essential to normal growth and metabolism [7]. However, under some pathological stimuli, such as I/R, the balance between free radical production and scavenging is broken and induces oxidative stress [8]. The accumulation of ROS damages cellular lipids, proteins, and DNA, which destroy their normal functions [9]. Mitochondria are a crucial site of ROS formation, and the excess of ROS destroys the function of mitochondria, thus inducing cell dysfunction [10]. Therefore, drugs which can scavenge the ROS and protect mitochondrial function may be useful for the treatment of I/R injuries.

Silent information regulator 1 (SIRT1), a histone deacetylase dependent on nicotinamide adenine dinucleotide (NAD+), regulates various cell functions including cell differentiation, survival, and metabolism [11]. SIRT1 deacetylates and activates forkhead box O (FOXO), which induces the expression of antioxidant proteins including manganese superoxide dismutase (MnSOD) and catalase (CAT), thus promoting the cellular resistance activity against oxidative stress [12]. Another target of SIRT1 is peroxisome proliferator-activated receptor coactivator 1-*α* (PGC-1*α*), a member of the family of transcriptional coactivators. PGC-1*α* not only induces the increase in mitochondrial number and intracellular ATP concentration but also induces the ROS-detoxifying enzymes which suppress ROS production [13].

*Aralia taibaiensis* (AT) is a Chinese medicinal herb, and its main constituents are triterpenoid saponin (sAT). It has been proven to have many effects, including antioxidative, antihyperlipidemic, antihyperglycemic, and antiapoptotic effects [14–17]. Clinically, it is always used to treat diabetes and related vascular diseases and depressive disorder, as well as ischemic brain and heart diseases [18, 19]. In previous studies, we showed that the antioxidative effects of sAT were better than those of the saponin from Panax ginseng, Schisandra chinensis, and other saponins [14]. However, few studies have investigated the protective effects and mechanisms of sAT on I/R-induced brain injuries. A previous study showed that sAT can promote the phosphorylation of Akt, upstream of SIRT1 and PGC-1*α* [20, 21]. Thus, in this study, we hypothesize that sAT protects brain I/R injury through the SIRT1/PGC-1*α*/FOXO3a pathway by activating Akt.

## 2. Materials and Methods

### 2.1. Materials

Dulbecco's modified Eagle's medium (DMEM) was obtained from HyClone (Logan, UT, USA). Fetal bovine serum (FBS) was provided by Hangzhou Sijiqing Biological Engineering Materials Co. Ltd. (Hangzhou, China). Annexin V-FITC apoptosis detection kit was obtained from Kaiji Biotechnology (Hangzhou, China). The kits for determination of LDH, caspase 3, DHE, DCF-DA, 8-Oxo-dG, protein carbonyl, MDA, SOD, CAT, GSH-Px, and GSH were obtained from Jiancheng Bioengineering Institute (Nanjing, China). MitoSOX Red and Rh123 staining were obtained from Molecular Probes (Eugene, OR). Antibodies for cleaved caspase 3, Bcl-2, Bax, cytochrome C, UCP2, SOD2, NOX4, COX IV, P-FOXO3a, PCNA, BIM, P27KIP1, CAT, NRF1, TFAM, P-PGC-1*α*, PGC-1*α*, SIRT1, P-Akt, Akt, FOXO3a, acetylated lysine, and *β*-actin were purchased from Cell Signaling Technology (Beverly, MA, USA). All other reagents were purchased from Chinese suppliers.

### 2.2. Cell Treatment

Mouse hippocampal neuron cell line HT22 was purchased from the Chinese Center for Type Culture Collection, Wuhan University, and cultured in DMEM containing 10% fetal bovine serum and antibiotics (penicillin, 100 IU/mL; streptomycin, 100 *μ*g/mL) at 37°C in 5% CO_2_ in a humidified incubator.

HT22 cells (1 × 104/well in a 96-well plate and 5 × 105/well in a 6-well plate) were pretreated with sAT (15, 30, and 60 *μ*g/mL) or NAC (positive control, 50 *μ*M) for 24 h and then incubated with Earle's balanced salt solution (116 mmol/L NaCl, 5.4 mmol/L KCl, 0.8 mmol/L MgSO_4_, 1 mmol/L NaH_2_PO_4_, 0.9 mmol/L CaCl_2_, and 10 mg/L phenol red) in a hypoxia chamber (Thermo Scientific, USA) containing a gas mixture of 95% N_2_ and 5% CO_2_ for 3 h. Then, cells were treated with the normal culture medium with or without sAT for 6 h to imitate the reperfusion process. Cells treated with the normal condition were used as control.

### 2.3. Analysis of Cell Viability

After different treatments, cell viability was measured by a Cell Counting Kit (CCK8) assay kit (ZETA Life Inc., Menlo Park, CA, USA) using a microplate reader (Bio-Rad Laboratory, Hercules, CA) 450 nm. The cell viability in every group was shown as the fold of control.

### 2.4. Determination of Apoptosis by Flow Cytometry Analysis

The rate of apoptosis of HT22 cells was measured by an Annexin V-FITC/PI apoptosis detection kit according to the manufacturer's instructions (Nanjing Jiancheng) using flow cytometer analysis (BD FACSAria II). A total of 10000 events were recorded in every sample.

### 2.5. Biochemical Analysis

HT22 cells were homogenized by RIPA buffer, and proteins were collected. Levels of LDH, caspase 3, 8-Oxo-dG, protein carbonyl, MDA, SOD, CAT, GSH-Px, and GSH were measured by commercially available kits as the manufacturer's protocol.

### 2.6. ROS Measurement

Levels of O_2_^·-^ in mitochondria were measured by MitoSOX Red which is a highly selective fluorescent probe for O_2_^·-^ generated within mitochondria. Levels of free radicals (H_2_O_2_) in cells were measured by 2,7-dichlorofluorescin diacetate (DCFH-DA). Levels of O_2_^·-^ in cells were measured by 2,7-dichlorofluorescin diacetate (DCFH-DA).

### 2.7. siRNA Transfection

SIRT1, Akt, FOXO3a, and PGC-1*α*-specific short interfering RNA (siRNA) molecules were chemically synthesized by Shanghai Genechem Company. Transfection was performed by utilizing Lipofectamine RNAiMAX reagent as per the manufacturer's protocol (Life Technologies, CA, USA). After 48 h transfection, cells were treated with sAT for 24 h and then subjected to I/R for another 9 h.

### 2.8. FOXO3a and PGC-1*α* Acetylation Assays

FOXO3a and PGC-1*α* lysine acetylation was analyzed by immunoprecipitation (IP) of FOXO3a and PGC-1*α* followed by Western blotting using antiacetylated lysine antibodies. FOXO3a and PGC-1*α* were immunoprecipitated using anti-FOXO3a and anti-PGC-1*α* antibodies, respectively. Acetylated FOXO3a and PGC-1*α* were detected using specific antibodies for antiacetylated lysine antibodies.

### 2.9. Animal Studies

Male Sprague Dawley rats (3 months, 200–230 g) were obtained from the Experimental Animal Center of the Fourth Military Medical University. All of the protocols in this study were approved by the Ethics Committee for Animal Experimentation and performed according to the Guidelines for Animal Experimentation of the Fourth Military Medical University and the National Institute of Health Guide for the Care and Use of Laboratory Animals (NIH Publication No. 80-23) revised in 2011.

Rats were randomly divided into five groups: sham, model, and sAT treatment groups (80 mg/kg, 160 mg/kg, and 320 mg/kg), 10 rats in every group. sAT was pretreated through intragastric administration for 7 days. Then, middle cerebral artery occlusion (MCAO) was induced by an intraluminal filament method as previously described [22]. After 1.5 h of MCAO, reperfusion was initiated by the withdrawal of the intraluminal suture.

### 2.10. Neurological Deficit Evaluation

A neurologic test was performed after reperfusion using a modified scoring system developed by Longa et al. [23]. Neurological function was graded on a scale of 0 to 5: 0, no deficits; 1 = failure to extend left forepaw fully, 2 = circling to the left, 3 = falling to the left, 4 = no spontaneous walking with a depressed level of consciousness, and 5 = dead.

### 2.11. Infarct Volume Assessment

Infarct size after MCAO was determined after different treatments using TTC staining. Brains were cut into slices of 2 mm thickness and incubated with 2% TTC solution for 20 min at 37°C. The volumes for each slice were then summed to determine the whole infarct volume of each brain.

### 2.12. Brain Water Content Measurement

Animals were anesthetized and sacrificed, and the brains were collected and weighed to get the wet weight (WW) and then dried at 110°C for 24 h to determine their dry wet (DW). The formula for calculating the water content was water content (%) = (WW − DW)/WW × 100.

### 2.13. Western Blotting

Protein in the nucleus and cytoplasm was obtained using a protein extraction kit, and the concentration was determined with a BCA protein assay reagent kit. 30 *μ*g of protein was resolved on 8–12% sodium dodecyl sulfate-polyacrylamide gel electrophoresis (SDS-PAGE) transferred onto polyvinylidene difluoride (PVDF) membranes. The membranes were blocked with 5% nonfat dry milk for 2 h at room temperature and then incubated overnight at 4°C with the following primary antibodies: cleaved caspase 3, Bcl-2, Bax, cytochrome C, UCP2, SOD2, NOX4, COX IV, P-FOXO3a, PCNA, BIM, P27KIP1, CAT, NRF1, TFAM, P-PGC-1*α*, PGC-1*α*, SIRT1, P-Akt, Akt, FOXO3a, acetylated lysine, and *β*-actin. Then, membranes were washed and incubated with secondary antibodies for 1 h at room temperature and visualized by an enhanced chemiluminescent substrate (Thermo Fisher Scientific). The densities of membranes were scanned and quantified by image analysis systems (Bio-Rad, USA).

### 2.14. Statistical Analysis

Data from individual experiments were presented as mean ± SD. One-way ANOVA followed by the Tukey test was performed by using GraphPad Prism 5.0 (GraphPad Software, La Jolla, CA). *P* < 0.05 was considered to be statistically significant.

## 3. Results

### 3.1. sAT Protected Cells from I/R-Induced Cell Injury

**Figure 1:**
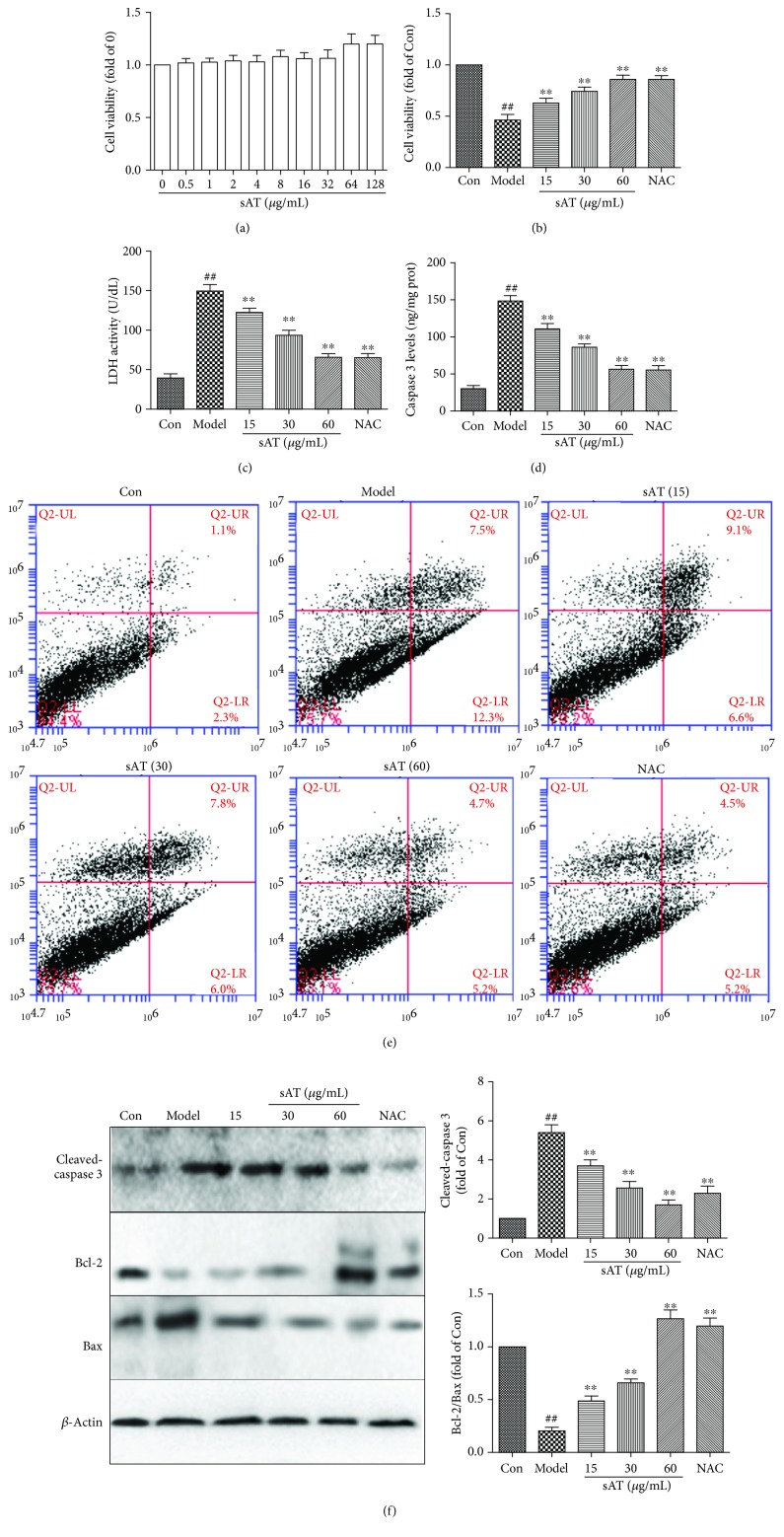
Effects of sAT on cell viability and apoptosis. (a) HT22 cells were treated with different doses of sAT for 24 h; then, the cell viability was measured by a CCK8 kit. (b). Cells were pretreated with sAT for 24 h and then treated with I/R for another 9 h. (c) LDH levels in cell culture media were measured by a LDH determination kit. (d) Caspase 3 levels in cells were measured by a caspase 3 determination kit. (e) After different treatments, the cell apoptosis rates were measured by an Annexin V-FITC/PI double-labeled kit using flow cytometry. (f) The protein levels of cleaved caspase 3, Bax, and Bcl-2 were measured by Western blotting. The columns and errors bars were represented as means ± SD (*n* = 3). ^##^*P* < 0.01 vs. the Con group, ^∗∗^*P* < 0.01 vs. the model group.

As shown in Figure 1(a), sAT had no cell toxicity to HT22 cells up to 128 *μ*g/mL, so 15, 30, and 60 *μ*g/mL which had lesser effects on cell viability were selected in the further studies. The cell viability was reduced to 46.3 ± 9.4% when treated with I/R for 9 h compared with the control group. Treatments of sAT exhibited a beneficial effect on cell viability and showed a dose-dependent manner (Figure 1(b)). LDH is a marker of cell injury; it will be released from the cells when the cells are injured by some factors. I/R treatment significantly increased the LDH levels in culture media, indicating the cytomembrane was broken by I/R. In sAT treatment groups, LDH levels were significantly decreased which was compared with the model group (Figure 1(c)).

We also detected apoptosis by caspase 3 determination kit, Annexin V-FITC/PI, and Western blotting. From the results shown in Figures 1(d)–1(f), I/R increased the caspase 3 levels and the apoptosis rate and decreased the Bcl-2/Bax ratio, indicating that I/R increased the cell apoptosis. Treatment with sAT significantly deceased the caspase 3 levels and apoptosis rate and increased the ratio of Bcl-2/Bax, showing the inhibition effects on I/R-induced cell apoptosis.

### 3.2. sAT Inhibited Oxidative Stress Induced by I/R

**Figure 2:**
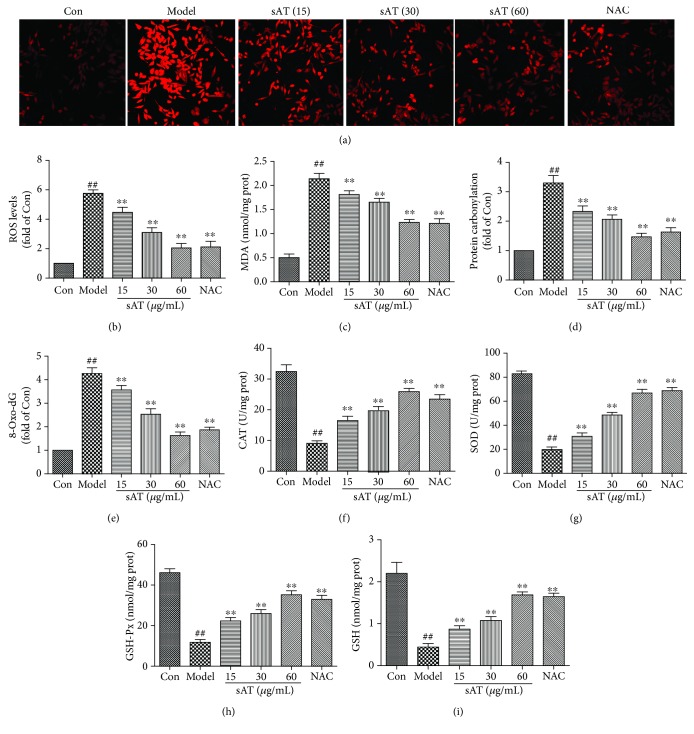
Effects of sAT on the oxidative stress induced by I/R. (a) Superoxide radical level was measured by a DHE kit using a laser confocal microscope (×200). (b) ROS levels were measured by a reactive oxygen species assay kit. The effects of sAT on the levels of MDA (c), protein carbonyl (d), 8-Oxo-dG (e), CAT (f), SOD (g), GSH-Px (h), and GSH (i) were measured as described in Materials and Methods. The columns and errors bars were represented as means ± SD (*n* = 3). ^##^*P* < 0.01 vs. the Con group, ^∗∗^*P* < 0.01 vs. the model group.

To investigate the effects of sAT on oxidative stress induced by I/R, ROS and related proteins were measured. ROS levels were measured by immunofluorescence using DHE dye and reactive oxygen species assay kit using DCF-DA. From the results shown in Figures 2(a) and 2(b), I/R induced significantly an increase in ROS levels. In the sAT treatment groups, ROS levels were decreased in a dose-dependent manner. Excess ROS will induce lipid, protein, and DNA oxidation. In Figures 2(c)–2(e), I/R increased the MDA, protein carbonyl, and 8-Oxo-dG levels significantly which were the markers of lipid, protein, and DNA oxidation production, respectively. Compared with the model group, the MDA, protein carbonyl, and 8-Oxo-dG levels were lowered in a dose-dependent manner (*P* < 0.01). Antioxidant enzymes were also measured by ELISA kits (Figures 2(f)–2(i)). I/R decreased the CAT, SOD, GSH-Px, and GSH levels in cells compared with control cells; however, sAT restored these protein levels to some extent (*P* < 0.01).

### 3.3. sAT Inhibited Mitochondrial Dysfunction Induced by I/R

**Figure 3:**
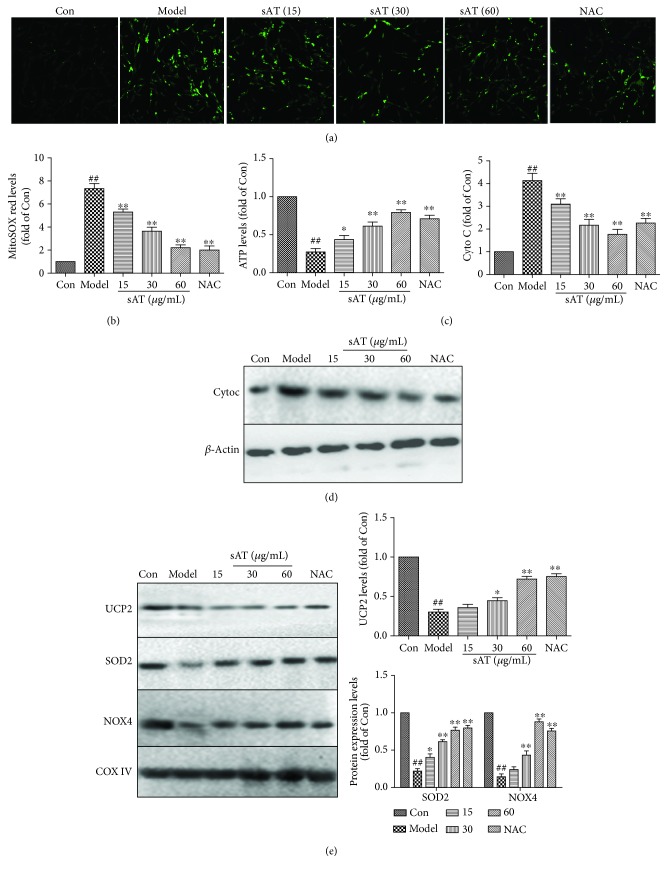
Effects of sAT on mitochondrial dysfunction induced by I/R. (a) Mitochondrial transmembrane potential (MMP) was detected by Rh123 staining. Laser confocal microscopy was used to take photos (×200). (b) Mitochondrial superoxide was detected by mitoSOX red staining. (c) ATP contents in cells were detected by an ATP determination kit. The protein expression levels in the cytoplasm (d) and mitochondrial fraction (e) were detected by Western blotting. The columns and errors bars were represented as means ± SD (*n* = 3). ^##^*P* < 0.01 vs. the Con group. ^∗^*P* < 0.05, ^∗∗^*P* < 0.01 vs. the model group.

Mitochondrial transmembrane potential (MMP) was detected by Rh123 staining. From the results shown in Figure 3(a), the green fluorescence in the model group was much higher than that in the control group, indicating that the MMP was destroyed by I/R treatment. In sAT treatment groups, especially in the 60 *μ*g/mL treatment group, green fluorescence was significantly decreased. A significant elevation (*P* < 0.01) of mitoSOX fluorescence (an indicator of mitochondrial superoxide) was detected in I/R-treated cells and they were suppressed to a considerable extent by pretreatment with sAT (Figure 3(b)). As shown in Figure 3(c), ATP detection results indicated that sAT treatment ameliorated the injured mitochondrial respiratory function following I/R. Western blotting results of cytochrome C in Figure 3(d) showed that cytochrome C content in the cytoplasm was higher in the I/R treatment group compared with the control group. In sAT treatment groups, the cytosolic expressions of cytochrome C were remarkably decreased in a dose-dependent manner. I/R treatment also induced a significant decrease in UCP2, SOD2, and NOX4 expression in mitochondrial fraction (Figure 3(e)). sAT pretreatment significantly increased the expression of UCP2, SOD2, and NOX4. These results showed that sAT protected cells from I/R-induced mitochondrial dysfunction.

### 3.4. sAT Upregulated the Expression of FOXO3a and Its Downstream Proteins

**Figure 4:**
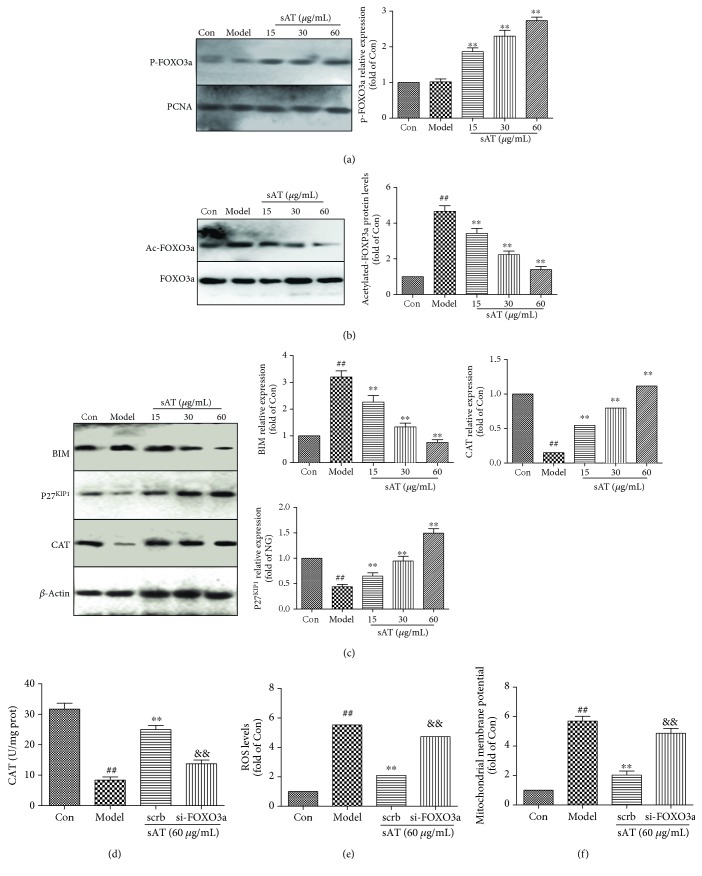
The effects of sAT on the FOXO3a pathway. (a) The phosphorylation of FOXO3a in the nucleus was measured by Western blotting. (b) Ac-FOXO3a was immunoprecipitated using a FOXO3a antibody. (c) The downstream proteins of FOXO3a (CAT, P27^KIP1^, and BIM) were measured by Western blotting. HT22 cells were transfected with FOXO3a siRNA for 48 h, then treated with sAT (60 *μ*g/mL) for 24 h and I/R for another 9 h. The levels of CAT (d), ROS (e), and MMP (f) in cells were measured using relative measurement kits. The columns and error bars were represented as means ± SD (*n* = 3). ^##^*P* < 0.01 vs. the Con group, ^∗∗^*P* < 0.01 vs. the model group, and ^&&^*P* < 0.01 vs. the scrb group.

It has been reported that FOXO3a has an important role in fighting against oxidative stress. From the results shown in Figure 4(a), I/R had little influence on the phosphorylation level of FOXO3a in the nucleus; however, sAT promoted the phosphorylation of FOXO3a in the nucleus. The acetylation level of FOXO3a was also measured by immunoprecipitation. The results in Figure 4(b) showed that I/R induced FOXO3a acetylation significantly (*P* < 0.01) and sAT deacetylated FOXO3a in a dose-dependent manner. The Western blotting results showed that the downstream proteins of FOXO3a (P27^KIP1^ and CAT) were inhibited by I/R treatment and the BIM expression level was increased significantly (*P* < 0.01). In sAT treatment groups, P27^KIP1^ and CAT were increased and BIM was decreased to some extent (Figure 4(c)). To confirm the role of FOXO3a, siRNA was used to knock down FOXO3a. Compared with the scrb group, si-FOXO3a abolished the effects of sAT on CAT (Figure 4(d)), ROS (Figure 4(e)), and MMP (Figure 4(f)) levels.

### 3.5. sAT Upregulated the Expression of PGC-1a and Its Downstream Proteins

**Figure 5:**
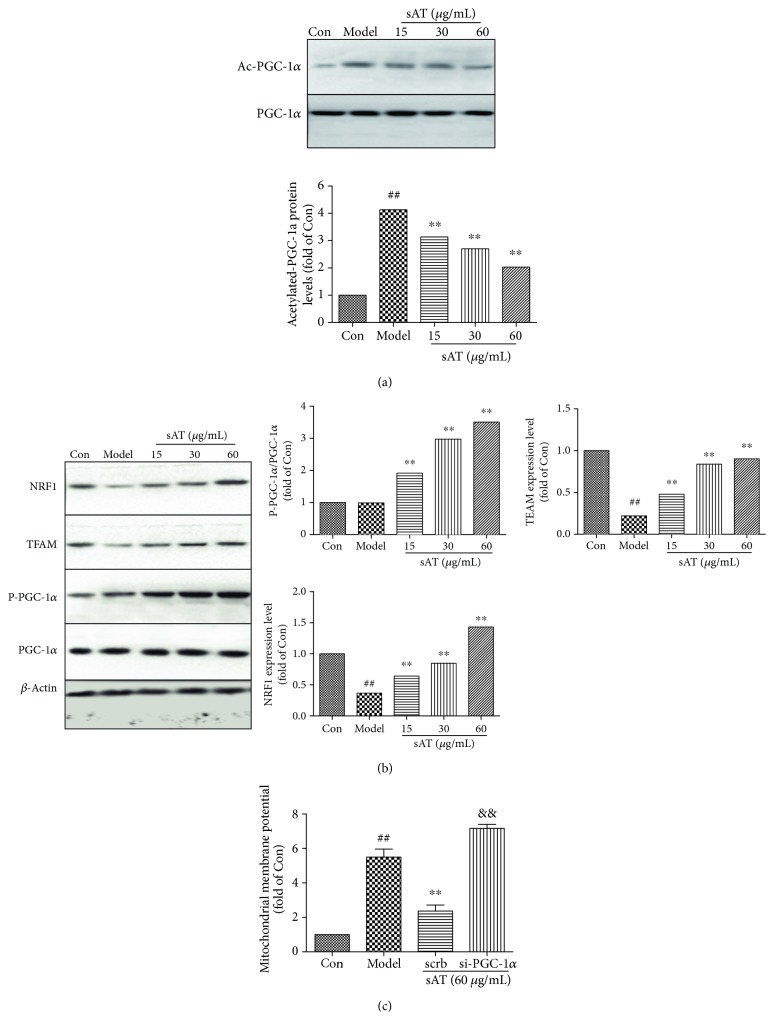
SAT protected mitochondrial function through the PGC-1a pathway. (a) Ac-PGC-1a levels were measured by immunoprecipitation. (b) The protein expression levels of NRF1, TFAM, P-PGC-1a, and PGC-1a were measured by Western blotting. (c) HT22 cells were transfected with PGC-1a siRNA for 48 h, then treated with sAT (60 *μ*g/mL) for 24 h and I/R for another 9 h. The mitochondrial membrane potential was measured by Rh123 staining. The columns and error bars were represented as means ± SD (*n* = 3). ^##^*P* < 0.01 vs. the Con group, ^∗∗^*P* < 0.01 vs. the model group, ^&&^*P* < 0.01 vs. the scrb group.

PGC-1a and its downstream proteins play an important role in I/R-induced injuries. From the results shown in Figure 5(a), I/R caused the increase in PGC-1a acetylation level, whereas sAT induced reduction in PGC-1a acetylation level compared with the model group (*P* < 0.01). The Western blotting results also showed that the phosphorylation levels of PGC-1a were increased by sAT treatment, as well as downstream (NRF1 and TFAM), indicating that sAT activated the PGC-1a pathway (Figure 5(b)). Further siRNA experiments showed that the mitochondrial membrane potential was destroyed by si-PGC-1a (Figure 5(c)), indicating that sAT protected the mitochondrial function through the PGC-1a pathway partly.

### 3.6. The Effects of sAT on FOXO3a and PGC-1*α* Were through SIRT1

**Figure 6:**
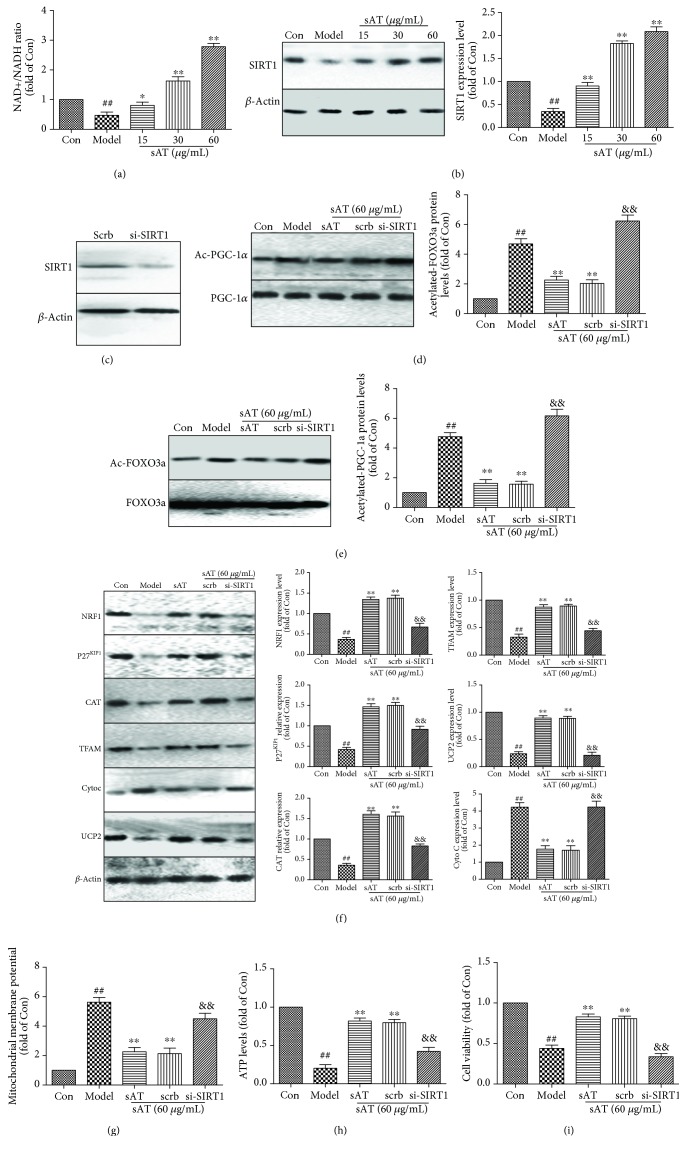
Effects of sAT were through upregulating SIRT1. (a) After different treatments, NAD+ and NADH levels were measured by a kit, then NAD+/NADH was calculated as direction. (b) The protein expression levels of SIRT1 were measured by Western blotting. HT22 cells were transfected with SIRT1-specific siRNA (Si-SIRT1) or control siRNA (scrb) for 48 h and treated with sAT (60 *μ*g/mL) with or without I/R treatment. (c) Effects of si-SIRT1 transfection on the expression levels of SIRT1. (d) Acetylated-PGC-1*α* (Ac-PGC-1*α*) was immunoprecipitated using the PGC-1*α* antibody. (e) Acetylated-FOXO3a (Ac-FOXO3a) was immunoprecipitated using the FOXO3a antibody. (f) After treatments with sAT and siRNA, NRF1, P27^KIP1^, CAT, TFAM, Cyto C, and UCP2 protein expression levels were measured by Western blotting. The effect of sAT and SIRT1 siRNA pretreatment on mitochondrial membrane potential (g), ATP levels (h), and cell viability (i). The columns and error bars were represented as means ± SD (*n* = 3). ^##^*P* < 0.01 vs. the Con group, ^∗∗^*P* < 0.01 vs. the model group, ^&&^*P* < 0.01 vs. the scrb group.

As the main deacetylase in cells, SIRT1 modulates various proteins to protect the cells from mitochondrial dysfunction and oxidative stress. FOXO and PGC-1*α* are both regulated by acetylation. To test whether sAT had a direct effect on SIRT1, NAD+/NADH levels and protein expression of SIRT1 were detected. NAD+/NADH levels and SIRT1 protein expression levels were decreased in I/R-treated cells, whereas sAT treatment significantly increased their levels compared with the model group (Figure 6(a) and 6(b)). To further illuminate the role of SIRT1, siRNA-targeted SIRT1 transfection was used (Figure 6(c)). From the results shown in Figures 6(d) and 6(e), compared with the scrb group, si-SIRT1 abolished the deacetylation effects of sAT on FOXO3a and PGC-1*α*. We also examined the expression of P27^KIP1^ and CAT, downstream target of FOXO3a, and NRF1 and TFAM, downstream target of PGC-1*α*. The results showed that P27^KIP1^, CAT, NRF1, and TFAM protein expression levels were inhibited by si-SIRT1 transfection (Figure 6(f)). Effects of sAT on mitochondrial function which were reflected by cytochrome C, UCP2 protein expression levels, mitochondrial membrane potential (Figure 6(g)), and ATP levels (Figure 6(h)) were also inhibited by si-SIRT1 transfection. In addition, the results of neuronal viability (Figure 6(i)) indicated that si-SIRT1 transfection partially reversed the sAT-induced protection against I/R injury.

### 3.7. sAT Upregulated SIRT1 through Akt

**Figure 7:**
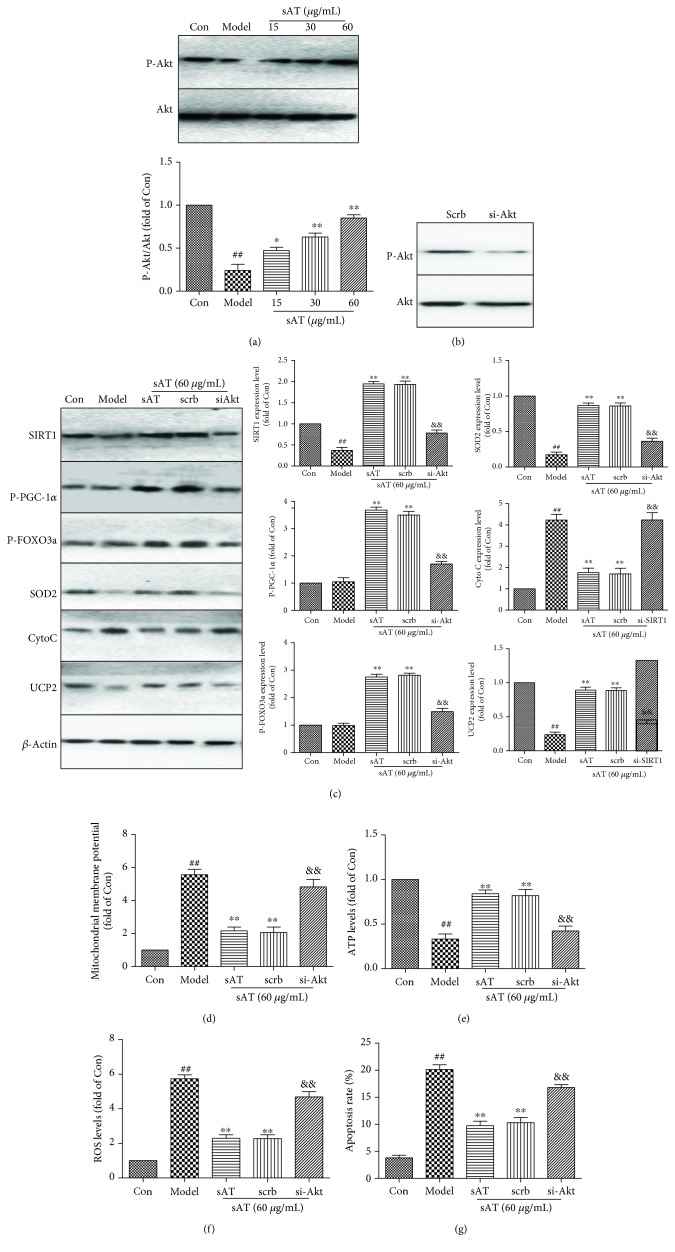
sAT activated SIRT1 via activation of PI3K/AKT signaling pathways. (a) Relative levels of phosphorylated Akt in HT22 cells pretreated with sAT followed by I/R for 24 h. HT22 cells were transfected with Akt-specific siRNA (si-Akt) or control siRNA (scrb) for 48 h and treated with sAT (60 *μ*g/mL) with or without I/R treatment. (b) Relative levels of phosphorylated Akt in HT22 cells after transfection. (c) The protein levels of SIRT1, P-PGC-1*α*, P-FOXO3a, Cyto C, SOD2, and UCP2 in HT22 cells after transfection. (d) Mitochondrial membrane potential was detected in cells after transfection. (e) ATP levels were measured as in the method shown in Materials and Methods. (f) ROS levels in HT22 cells after transfection. (g) Apoptosis rate in HT22 cells after transfection. The columns and error bars were represented as means ± SD (*n* = 3). ^##^*P* < 0.01 vs. the Con group, ^∗∗^*P* < 0.01 vs. the model group, ^&&^*P* < 0.01 vs. the scrb group.

To investigate the mechanism of sAT-induced SIRT1 activation, the Akt pathway was studied further. From the results shown in Figure 7(a), the phosphorylation status of Akt was decreased in the model group and increased in the sAT treatment groups, indicating that sAT had an activation effect on the Akt pathway. To investigate the relationship between Akt and SIRT1, siRNA-targeted Akt was used. The phosphorylation level of Akt was decreased significantly in siRNA-transfected cells (Figure 7(b)), accompanied by decreased SIRT1 expression and phosphorylation levels of PGC-1*α* and FOXO3a (Figure 7(c)). SOD2 and UCP2 protein levels were decreased and Cyto C levels were increased in the si-Akt group compared with the scrb treatment group, together with the results in mitochondrial membrane potential (Figure 7(d)) and ATP levels (Figure 7(e)), indicating that the mitochondrial functions were damaged by si-Akt treatment. si-Akt transfection also abolished the effects of sAT on ROS levels (Figure 7(f)) and apoptosis rates (Figure 7(g)). Hence, sAT exerted its effect mainly through the PI3K/AKT signaling pathway.

### 3.8. sAT Protected Brain Injury through Akt/SIRT1/FOXO3a/PGC-1*α* In Vivo

**Figure 8:**
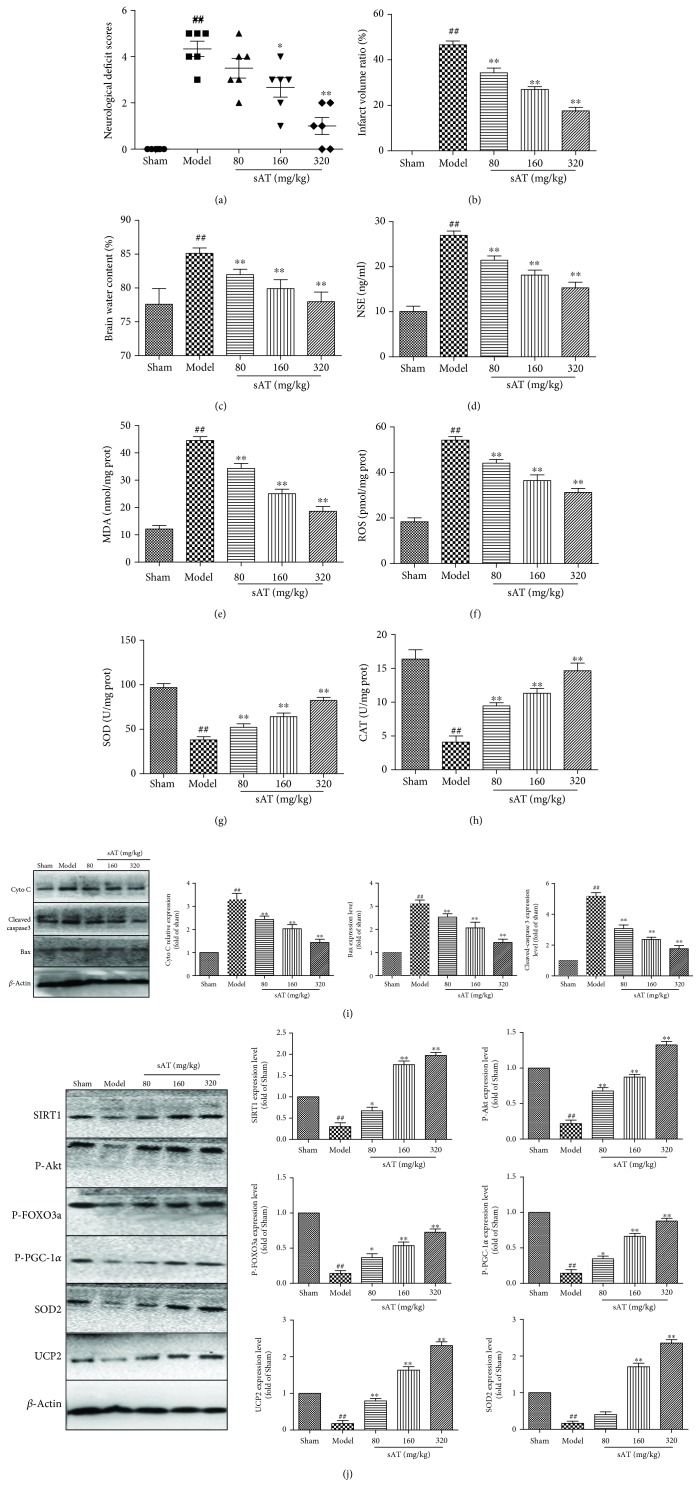
The Akt/SIRT1/FOXO3a/PGC-1*α* pathway was involved in the effects of sAT in a rat model. (a) Neurological scores in rats after stroke. (b) TTC staining was performed to evaluate the infarct formation after stroke. (c) The water content of ipsilateral hemispheres was measured to evaluate the degree of cerebral edema. (d) Levels of NSE in serum were detected by relative kits. The brain tissues were collected after different treatments and homogenized to determine the levels of MDA (e), ROS (f), SOD (g), and CAT (h). (i) Bax, Cyto C, and cleaved caspase 3 were detected by Western Blot as mentioned in Materials and Methods. (j) Effects of sAT on the expression levels of SIRT1, P-Akt, P-PGC-1*α*, P-FOXO3a, SOD2, and UCP2 in the cytoplasm. *β*-Actin was used as the internal control protein in the cytoplasm. The columns and error bars were represented as means ± SD (*n* = 3). ^##^*P* < 0.01 vs. the sham group; ^∗^*P* < 0.05, ^∗∗^*P* < 0.01 vs. the model group.

To study the protective effects of sAT *in vivo*, a MCAO and reperfusion rat model was used. Compared with the sham group, the neurological deficits (Figure 8(a)), infarct volume ratio (Figure 8(b)), brain water content (Figure 8(c)), and NSE levels (Figure 8(d)) were increased significantly in the MCAO and reperfusion treatment groups, which were decreased significantly in the sAT treatment groups. Oxidative stress was also observed in MCAO and reperfusion rat models, which is reflected by the increased levels of MDA (Figure 8(e)) and ROS (Figure 8(f)) and the decreased levels of SOD (Figure 8(g)) and CAT (Figure 8(h)). SAT significantly increased the antioxidant protein expression and decreased the ROS levels in a dose-dependent manner. MCAO and reperfusion also increased the apoptosis-related protein levels (Cyto C, cleaved caspase 3, and Bax), which were reduced by sAT in a dose-dependent manner (Figure 8(i)).

To determine whether the Akt/SIRT1/FOXO3a/PGC-1*α* pathway was involved *in vivo*, the protein levels were measured by Western blotting. From the results shown in Figure 8(j), the protein expression levels of SIRT1, P-Akt, P-PGC-1*α*, P-FOXO3a, SOD2, and UCP2 were decreased in the model group. However, in the sAT treatment groups, the levels of these proteins were increased significantly compared with the model group. These results indicated that sAT had protective effects against MCAO- and reperfusion-induced brain injuries in vivo; the Akt/SIRT1/FOXO3a/PGC-1*α* pathway may play an important role during this process.

## 4. Discussion

Ischemic stroke has been a leading cause of death and disability around the world. Because of the lack of effective therapies, studying ischemic stroke and its treatment means is still a major medical problem. Ischemia and following reperfusion will induce excessive accumulation of reactive oxygen species (ROS) in the brain which activate several signaling pathways and result in oxidative damage [24]. Accumulating evidence indicated that oxidative stress has been connected with the pathophysiology of neurotoxicity after brain ischemia [25]. Lots of literatures had shown that antioxidants protect the brain from injuries induced by brain ischemia and reperfusion (I/R) [26, 27]. Traditional Chinese herbs have been used to treat brain injuries, including stroke, for thousands of years. The main constituents in many herbs which contribute to their bioactivities have been identified with modern technologies [28]. Triterpenoid saponins have been proven to have many effects including anti-inflammatory, antioxidation, hypolipidemic, hypoglycemic, and antitumor as well as regulation of the immune system [29]. The main constituents in sAT are triterpenoid saponins and showed excellent effects in treating diabetes and its related vascular diseases. In previous studies, we also found that sAT attenuated D-galactose-induced rat aging and high glucose-induced myocardial injuries through its antioxidative effects [20, 30]. However, whether sAT has an ability to protect I/R-induced brain injury through its antioxidant properties is largely unclear. In this study, the effects of sAT on the I/R-induced brain injury and its possible mechanisms were studied in vitro and in vivo.

The first series of experiment results demonstrated that sAT protected brain cells against oxidative injury. In vitro, I/R induced the decrease in cell viability and increased the levels of caspase 3, LDH, and apoptosis, whereas sAT reversed these injuries and restored the cell viability. In vivo, sAT effectively reduced the brain infarct size, brain water content, and NSE levels and ameliorated neurological deficits which are induced by MCAO and reperfusion. The results of ROS and antioxidant protein detection showed that sAT increased the levels of antioxidant proteins and decreased the levels of ROS in HT22 cells and brain tissues in a dose-dependent manner. These results indicated that sAT protected brain cells from I/R-induced injuries and might be dependent on its antioxidative effects.

More and more studies show that mitochondrial dysfunction occurs in many brain diseases, such as Parkinson's disease and stroke [31, 32]. A majority of ROS in cells are produced by mitochondria, and the injuries induced by I/R are closely associated with mitochondrial ROS induction [33]. Excessive ROS levels also induce mitochondrial dysfunction and the release of cytochrome C which activates the mitochondrial apoptosis process [34]. In this study, we found that MMP, mitochondrial superoxide, and cytochrome C were increased by I/R treatment, together with the decrease in ATP production, indicating that I/R destroyed the function of mitochondria. In sAT treatment groups, these changes were reversed in a dose-dependent manner. The mitochondrial function-related protein and UCP2, SOD2, and NOX4 expression in mitochondrial fraction were also restored by sAT. These results indicated that sAT protected cells from I/R-induced mitochondrial dysfunction.

To our knowledge, PGC-1*α* is known to be a master regulator of mitochondrial function, cell metabolism, and oxidative stress [35]. The increase in PGC-1*α* expression in cells accelerates the recovery of mitochondrial and cellular functions to defend the injuries induced by some factors like I/R [13]. In brain cells, higher expression levels of PGC-1*α*reduced the neuronal death mediated by oxidative stress by increasing the expression of SOD2, a major antioxidant enzyme of mitochondria [36]. In mitochondria, PGC-1*α* induces the expression of mitochondrial genes and increases their activity, thus governing mitochondrial biogenesis and function [37]. The activity of PGC-1*α* is regulated by posttranslational modifications, including acetylation or phosphorylation [38]. In this study, the deacetylation and phosphorylation levels were both increased by sAT treatment, as well as downstream (NRF1 and TFAM), indicating that sAT activated the PGC-1*α* pathway in HT22 cells. In addition, si-PGC-1a transfection abolished the protective effects of sAT on mitochondrial function.

The FOXO proteins play as crucial regulators in various cellular processes including oxidative stress, apoptosis, and cell cycle progression [39]. Among the four known FOXO members, FOXO3a upregulates several antioxidant proteins including SOD and CAT and has an important role during oxidative stress [40]. FOXO3a is also known to induce the expression of proapoptotic proteins such as BIM and Fas-L [41]. The transcriptional activity of FOXO3a is regulated by deacetylation and phosphorylation [42]. Upon stimulation, FOXO3a is phosphorylated and binds to the 14-3-3 proteins in the nucleus, thus inhibiting FOXO3a-dependent transcription [43]. In the present study, the increase in the phosphorylation and deacetylation of FOXO3a was observed in HT22 cells, and the downstream proteins of FOXO3a (P27^KIP1^ and CAT) were increased and BIM expression levels were decreased. In addition, si-FOXO3a transfection abolished the protective effects of sAT on ROS production and mitochondrial function.

Next, we wanted to investigate the signaling pathway by which sAT regulated FOXO3a and PGC-1a. SIRT1 is a kind of nicotinamide adenine dinucleotide- (NAD+-) dependent histone deacetylase and plays an important role in regulating stress response [44]. SIRT1 has been shown to activate PGC-1a directly by deacetylation in different domains and thus induces the metabolic gene transcription rates controlling expression [45, 46]. SIRT1 has also shown to regulate mammalian FOXO transcription factors directly by deacetylation in the nucleus, thus increasing the ability of FOXO3a to induce antioxidant protein expression, cell cycle arrest, and DNA repair, while inhibiting the ability of FOXO3a to induce apoptosis [12, 47, 48]. Considering these, we assumed that the effects of sAT on FOXO3a and PGC-1a might through SIRT1. In this study, administration of sAT significantly increased the level of NAD+/NADH and the expression of SIRT1. In the further siRNA researches, we found that the deacetylation effects of sAT on FOXO3a and PGC-1*α* and the protective effects were abolished by si-SIRT1 transfection. These results confirmed our hypothesis that the effects of sAT on FOXO3a and PGC-1*α* were through SIRT1. Considering the effects of sAT on NAD+/NADH, other sirtuins such as SIRT3, SIRT4, and SIRT5 may be modulated by sAT, thus inducing mitochondrial biosynthesis and antioxidant enzyme expression to combat injuries caused by I/R.

The PI3K/Akt pathway plays very important roles in promoting cell survival by phosphorylating its downstream proteins in numerous cell systems [49]. Reports have documented that Akt can phosphorylate FOXO3*α* and PGC-1*α* thereby reducing brain injuries in a rat stroke model [50, 51].

The relationship between Akt and SIRT1 was also well studied in previous studies [52]. To investigate whether SIRT1 modulation by sAT depends on the Akt pathway, the phosphorylation level of Akt was detected by Western blotting. The present study results showed that sAT induced the phosphorylation of Akt in a dose-dependent manner, and the effects of sAT on the phosphorylation levels of PGC-1*α*, FOXO3a, and SIRT1 expression levels were abolished by si-Akt. These results suggested that sAT-induced SIRT1 expression was mediated by the Akt pathway.

In conclusion, we have demonstrated for the first time that sAT protected brain cells against I/R-induced mitochondrial dysfunction and oxidative stress through regulating the Akt/SIRT1/FOXO3a/PGC-1*α* pathway. These results might improve our understanding of the molecular mechanisms involved in sAT-induced cerebral protection effects.

## Data Availability

The data used to support the findings of this study are available from the corresponding author upon request.
